# Diagnosis and Management of Intrascrotal Nerve Tumors: A Systematic Review of the Literature

**DOI:** 10.5152/tud.2023.23050

**Published:** 2023-09-01

**Authors:** Ilias Giannakodimos, Alexis Giannakodimos, Afroditi Ziogou, Konstantinos Tzelepis

**Affiliations:** 1 Department of Urology, Geniko Kratiko Nikaias General Hospital, Athens, Greece; 2 Department of Medical Oncology, Metaxa Cancer Hospital, Athens, Greece

**Keywords:** Scrotal tumors, nerve tumors, cancer, systematic review

## Abstract

Scrotal tumors of nerve origin are extremely rare and occur mostly in the extratesticular tissues of scrotum, such as the spermatic cord and epididymis. A systematic search of the literature in PubMed, Medline, and Google Scholar databases concerning intrascrotal nerve tumors was performed by 2 independent investigators. The systematic search retrieved 45 male adults, with a mean age of included patients at 43.9 ± 18.8 years. The majority of nerve tumors were extra-testicular (86.7%), and only 13.3% originated from the testis. Out of that, 51.1% of neoplasms were histologically proved as schwannomas, 44.4% as neurofibromatosis, and 4.4% as malignant peripheral nerve sheath tumors. The majority of patients presented with atypical symptoms such as scrotal swelling (51.1%), while only 4.4% of patients were asymptomatic. Ultrasonography is the diagnostic modality of choice (97.2%) for the detection of primary lesion, while magnetic resonance imaging and computed tomography comprise supplementary diagnostic tools. Surgical excision of the mass was the preferred type of surgery performed (75.6%), whereas orchiectomy was performed only in 22.2% of patients. Intrascrotal tumors of nerve origin are extremely rare neoplasms that present mainly in middle-aged males. Increased clinical suspicion is required for accurate diagnosis of this rare entity.

**Main Points**

- The majority of scrotal tumors with nerve origin were extra-testicular (86.7%) and only 13.3% originated from the testis.
- 51.1% of neoplasms were histologically proved as schwannomas, 44.4% as neurofibromatosis and 4.4% as malignant peripheral nerve sheath tumors.
  Surgical excision of the mass was the preferred type of surgery performed (75.6%)
- Orchiectomy was performed only in 22.2% of patients.

## Introduction

Mesenchymal scrotal tumors are extremely rare and occur mostly in the extratesticular tissues of scrotum, such as the spermatic cord and epididymis.^1^ These neoplasms mainly include leiomyomas, fibromas, lipomas, hemangiomas, and nerve tumors that are either benign or malignant.^1^ Neurofibromas and schwannomas constitute the most frequently reported scrotal mesenchymal tum ors of the nerve.^1^ These neoplasms usually develop in various anatomic locations, especially in organs with abundant nerve supply, such as the neck and thorax, while their occurrence in the scrotum is considered extremely rare.^2^ Neurofibromas can present either as a solitary mass or as numerous lesions in the context of a systematic condition named Neurofibromatosis type 1 (NF1 or Von Recklinghausen's disease). Neurofibromatosis type 1 is an autosomal dominant disease characterized by disrupted development of neural crest cells and is associated with the development of various malignant neoplasms.^3,4^ Schwannomas are neuronal tumors that originate from Schwann cells. Although, in the majority of cases, schwannomas comprise benign entities, malignant transformation of these neoplasms has also been reported.^5^ Of note, nerve sheath tumors require thorough consideration concerning the clinical approach of patients with scrotal masses due to their potential for recurrence or malignancy, especially when associated with neurofibromatosis type 1.^6^ Only a few case reports of intrascrotal nerve sheath tumors have been reported in the literature, and therefore, their diagnosis mainly relies on the high suspicion of the urologist. The aim of the present study was to systematically review the literature concerning this rare entity and highlight proper management and optimal treatment of these neoplasms.

## Material and Methods

This systematic review was performed according to the Preferred Reporting Items for Systematic Reviews and Meta-Analyses guidelines.^7^ Two investigators (A.G. and A.Z.) searched independently PubMed/Medline and Google Scholar databases for eligible articles reporting on testicular, extra-testicular, and intrascrotal nerve tumors, especially neurofibromatosis and schwannoma, until November 13, 2022. The following keywords combined with the terms AND/OR were used for the search strategy: “testicular,” “extratesticular,” “intrascrotal,” “neurofibromatosis,” “schwannoma,” and “nerve tumor.” Any dispute was resolved by the intervention of a senior investigator (I.G.). This systematic review included case reports and case series that referred to testicular, extratesticular, and intrascrotal nerve tumors in males and were written only in English language. However, reviews and systematic reviews were excluded. Inaccessible articles, letters to the editor, comments, articles “epub ahead of print,” and studies referring to animal reports had to be excluded from the systematic review. Studies with no clear diagnosis or insufficient data, or articles referring to younger patients were excluded. Moreover, an additional search of the references of the eligible articles was performed in order to assess potential studies following the snowball procedure.

Three investigators (I.G., A.G., and A.Z.) worked independently and extracted information from all the studies included in this systematic review, using a pre-designed template. Data concerning age, tumor location, symptoms, and medical history of the patients was accumulated. The researchers also compiled information regarding physical examination, imaging features, histological findings, type of surgery undergone, and follow-up.

### Statistical Analyses

Numerical variables were presented as mean ± SD (standard deviation) or as median (25%-75% quartiles), in case they were skewed. Categorical variables were presented using frequencies and percentages. Patients included in case series were considered as unique case reports in order to estimate variables of interest. Several studies did not report on all outcomes of interest and therefore relative rates were estimated based on available data. No statistical relationship between the included variants was tested. Statistical analysis was carried out using IBM Statistical Package for the Social Sciences Statistics for Windows, version 24.0 (IBM SPSS Corp., Armonk, NY, USA).

## Results

**Figure 1.**
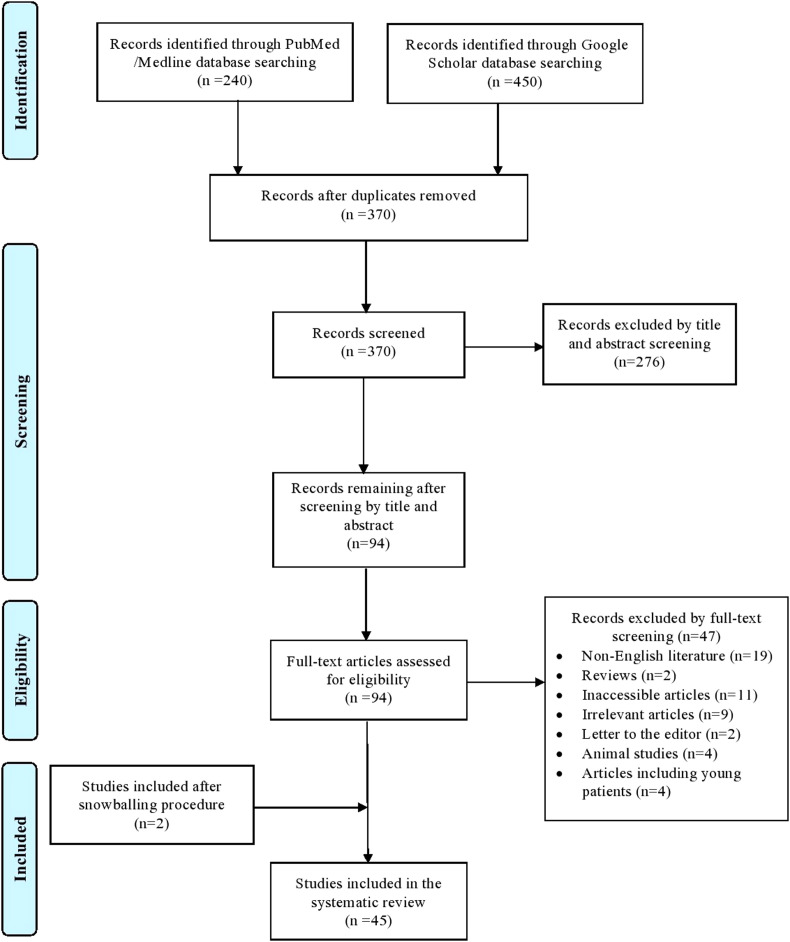
Trial flow of this systematic review.

The literature search retrieved 690 studies. After the exclusion of the duplicate studies, the record screening, and the snowball procedure, only 45 articles, published from 1939 to 2021, met the inclusion criteria and were included in the systematic review. A flow diagram of the selection process is depicted in Figure 1.

The selected articles included 45 male adults in total, and the mean age of patients with neurotic scrotal tumors was 43.9 ± 18.8 years (mean, SD). Out of the included studies, 51.1% (23 cases) of neoplasms were histologically proved as schwannomas, 44.4% (20 cases) as neurofibromatosis, and 4.4% (2 cases) as malignant peripheral nerve sheath tumors. The majority of intrascrotal tumors with neurogenic origin were extra-testicular (39, 86.7%), while only in 6 patients (13.3%) the primary tumor originated from the testis. Concerning anatomic origin, in 35 patients (77.8%), tumor originated from the scrotal parenchyma in 3 patients in the testis (6.6%), in 5 patients in the spermatic cord (11.1%), in 3 patients in tunica albuginea (6.7%), and in 1 patient in the epididymis (2.2%). Interestingly, only 1 patient (2.2%) had a medical history of neurofibromatosis type,**^8^** while 48 patients (97.8%) presented with no history of neurofibromatosis syndrome.

The majority of the included patients complained of scrotal swelling (23 patients, 51.1%), while only 2 patients (4.4%) were asymptomatic. Moreover, 15 patients (33.3%) presented with a painless lump in the scrotum area, 4 patients (8.9%) with scrotal discomfort, and 4 patients (8.9%) with scrotal pain. Other reported symptoms included penile pain, disability of erection, and ulceration of the scrotum. Detailed clinical manifestations and signs of the included patients are demonstrated in Table 1. Out of the available data, the mean duration of symptoms was estimated at 31.22 ± 44.4 months. Maximum duration of symptoms was estimated at 15 years, while only 3 patients presented with acute symptomatology that required immediate treatment.

Out of the 36 cases that presented with data o imaging findings, ultrasonography (US) was utilized in 35 patients (97.2%) aiming to detect the primary lesion, magnetic resonance imaging (MRI) in 6 patients (16.7%), computed tomography (CT) in 5 patients (12.9%), and positron emission tomography-computed tomography in 1 patient (2.6%). The ultrasound characteristics of intrascrotal nerve tumors are summarized in Table 2.

Surgical treatment comprised the optimal therapeutic approach in the included patients, and surgical excision of the mass was the preferred type of surgery (34 patients, 75.6%). Furthermore, orchiectomy was performed in 10 patients (22.2%), epididymectomy in 1 patient (2.2%), and penectomy in 1 patient (2.2%). Lymph node dissection along with orchiectomy was performed in only 1 patient. Patient’s survival was reported only in 24 cases, and the follow-up interval ranged from 1 month to 72 years among studies. Νο death was reported during this follow-up. Finally, malignant transformation of the intrascrotal tumor was reported only in 3 cases (8.8%). The epidemiologic characteristics of the included case reports are summarized in Table 3.

## Discussion

Tumors of the peripheral nervous system constitute rare entities with heterogeneous spectrum of morphological features and biological potential.^9^ These neoplasms consist of benign lesions with low malignant potential that are curable after mass excision, such as schwannoma; potentially aggressive benign tumors, such as plexiform neurofibroma; and highly malignant neoplasms, such as malignant peripheral nerve sheath tumors.^9^ More specifically, Schwannomas constitute benign nerve sheath neoplasms composed of neoplastic Schwann cells. Furthermore, neurofibromas are benign tumors that consist of neoplastic Schwann cells that also contain various non-neoplastic entities, such as fibroblasts, mast cells, perineurial-like cells, and residual axons. Schwannomas and neurofibromatosis are strongly related to the familiar syndromes NF2 and NF1, respectively.^9^ Schwannomas are usually found in the cranial nerves, brain, and vestibular branch of the VIII cranial nerve, while neurofibromas are predominantly found cutaneously. Their presence in the lower genitourinary tract, and especially in the scrotum area, remains extremely rare, and only few case reports have been described in the literature.^10^ To our knowledge, this is the first systematic review concerning nerve tumors of the genitals.

Intrascrotal masses that originate either from the testis or paratesticular tissues, such as scrotal parenchyma, epididymis, and spermatic cord, comprise common findings in the male population.^11^ Although testicular lesions are predominantly malignant, paratesticular masses are usually benign.^12^ Tumors commonly seen in the scrotum consist of leiomyomas, lipomas, fibromas, and hemagiomas.^11^ Nerve tumors of the intrascrotal area are extremely rare, and only few case reports have been reported in the literature. According to our systematic analysis, the mean age of patients was 43.9 years. The majority of tumors involved the extra-testicular area, while only 13.3% (6 patients) of the included patients involved the testis.

Patients with nerve tumors of the scrotum usually present with scrotal swelling along with a painless palpable mass or other atypical clinical manifestations. According to our systematic review, scrotal swelling (51.1%), existence of a painless mass (33.3%), and scrotal discomfort (8.9%) or pain (8.9%) comprise the most common clinical findings of this rare entity. Due to non-specific symptoms of these tumors, their diagnosis mainly depends on the clinical suspicion of physicians. Of note, the urologist should differentiate primary origin of the tumor, testicular or extra-testicular origin, by palpation since it affects optimal margins of the surgical excision. In our analysis, in 7 patients, radical orchiectomy and penectomy were mistakenly performed since these paratesticular tumors did not involve the testis or penis. As a result, a more conservative treatment, with solely excision of the mass, would be enough in these cases.

Concerning the diagnostic approach of intrascrotal nerve tumors, radiologic findings are usually non-specific and pose a diagnostic challenge for the physician.^13^ Ultrasonography can help in the differentiation between solid and cystic tumors, while CT scan may help in the better determination of tumor characteristics and its relation with the surrounding strictures.^13^ Magnetic resonance images provide similar locoregional information as CT but yield better visualization of the primary lesion.^13^ According to our systematic review, US was the most commonly utilized imaging test (97.2%) aiming to detect the primary lesion, while MRI and CT were performed in 16.7% and 12.9% of included patients, respectively. Despite the advancement of imaging modalities, the identification of scrotal nerve tumors remains challenging, and they should be differentiated from other clinical entities that could be developed in the intrascrotal area.

Although imaging techniques can aid in the initial identification of this pathologic entity, the ultimate diagnosis of these tumors relies on histopathological findings and immunohistochemical staining of resected specimens.^13^ Schwannomas usually present with a typical histologic pattern, including compact areas (Antoni A) of increased cellularity and spindle cells and loose areas (Antoni B) along with Verocay bodies.^9^ In addition, histologic findings of neurofibromas usually contain a predominance of Schwann cells embedded in a myxoid matrix with amounts of collagen fibers.^9^ Concerning immunohistochemical staining, both schwannomas and neurofibromas are characterized by the expression of S-100 and SOX-10 staining markers.^8,9^ However, in our study, we did not manage to perform a systematic record of staining markers for these entities due to the heterogeneity of included studies in staining techniques along with a restricted number of included cases that presented their immunohistochemical findings.

Surgical operations, either resection of the tumor or more radical surgical procedures, constitute the mainstay of treatment for intra-scrotal nerve tumors.^13^ The extent of surgical resection mainly depends on whether the mass involves the testis. In the majority of nerve tumors without testicular involvement, surgical resection of the mass with preservation of the testis comprises the optimal surgical approach. However, in our study, in 13.33% of the included cases without testis involvement, testicular resection was mistakenly performed. Of note, if the mass involves surrounding organs, radical tumor excision along with involved organ resection are considered mandatory. In our systematic review, resection of testis was performed in all cases of testicular involvement. Radical excision of the tumor is considered mandatory due to the possibility of malignant transformation or the malignant nature of the tumor. In our systematic analyses, 4.4% of included tumors were malignant, while in 8.8% of included cases, malignant degeneration of an initially benign tumor was found.

To the best of our knowledge, this is the first systematic review of the literature concerning epidemiology, clinical appearance, diagnostic approach, and therapeutic management of intrascrotal nerve tumors. Due to the scarcity of these tumors, extended eligibility criteria were used to include all types of nerve tumors developed in the scrotum of adult patients. However, our systematic analysis is subject to certain limitations. Our study included only case reports and case series with sufficient data, whose credibility mainly depends on accurate recordkeeping. In addition, the heterogeneity among institutions concerning surgical approaches and recordkeeping definitely affects outcomes and time-to-event analysis.

## Conclusion

Intrascrotal tumors of nerve origin are extremely rare neoplasms that are present mainly in middle-aged males and are not associated with a medical history of NF1. These tumors usually present with scrotal swelling along with other atypical symptoms, constituting a diagnostic challenge for physicians. The final diagnosis is confirmed by immunohistochemical staining along with specific histological evidence. Surgical resection of the malignancy, either with local excision or more radical procedures, constitutes the mainstay of treatment. Increased clinical suspicion is necessary for accurate diagnosis of this rare entity.

## Figures and Tables

**Table 1.** Clinical Manifestations of Intrascrotal Nerve Tumors

Symptoms	Patients (n = 43)	Percentage
Scrotal swelling	23	51.1%
Painless lump	15	33.3%
Scrotal discomfort	4	8.9%
Scrotal pain	4	8.9%
Tenderness	1	2.2%
Inguinal discomfort	2	4.4%
Scrotal ulceration	1	2.2%
Painful lump	1	2.2%
Penile pain	1	2.2%
Disability of erection	1	2.2%
Asymptomatic	2	4.4%

**Table 2.** Imaging Modalities for the Detection of Intrascrotal Nerve Tumors

Imaging Modalities	Patients (n = 36)	Percentage
US	35	97.2%
MRI	6	16.7%
CT	5	12.9%
PET-CT	1	2.6%

CT, computed tomography; MRI, magnetic resonance imaging; PET-CT, positron emission tomography-computed tomography; US, ultrasonography.

**Table 3.** Epidemiologic Characteristics of Included Case Reports

Authors	Year of Publication	Age of Patient	Surgery	Type of Surgery
Kim et al^14^	2013	67	Yes	Mass excision
Alsunbul et al^15^	2020	38	Yes	Mass excision
Arciola et al^16^	1985	53	Yes	Mass excision
Bergeron et al^17^	2014	16	Yes	Mass excision
Bian et al^2^	2020	43	Yes	Radical orchiectomy
Boto et al^18^	2015	38	Yes	Mass excision
Chan et al^13^	2007	28	Yes	Mass excision and partial scrotectomy
Gupta et al^6^	2010	24	Yes	Mass excision
Hosseini et al^1^	2011	52	Yes	Mass excision
Ikari et al^19^	2010	66	Yes	Mass excision
Bhanvadia et al^20^	2010	32	Yes	Mass excision
Issa et al^21^	1993	77	Yes	Mass excision
Kazarian et al^3^	2021	25	Yes	Radical penectomy
Levant et al^22^	1946	59	Yes	Radical orchiectomy
Milathianakis et al^23^	2001	86	Yes	Mass excision
Palleschi et al^8^	2014	52	Yes	Mass excision
Pinilla et al^24^	2009	60	Yes	Orchiectomy
Pujani et al^25^	2017	23	Yes	Mass excision
Safak et al^5^	1992	48	Yes	Scrotum removal
Shahid et al^26^	2014	45	Yes	Mass excision
Turkyilmaz et al^27^	2004	14	Yes	Mass excision
Livolsi et al^28^	1977	23	Yes	Radical orciectomy
Mahobia et al^29^	2018	55	Yes	Mass excision
Gkikas et al^30^	2016	24	Yes	Mass excision
Barde et al^31^	2013	24	Yes	Mass excision
Bhide et al^32^	2019	77	Yes	Radical orchiectomy
Mishra et al^33^	2002	33	Yes	Mass excision
Shamsa et al^34^	2004	57	Yes	Radical orchiectomy
Shukla et al^35^	2017	45	Yes	Mass excision
Singal et al^36^	2012	60	Yes	Mass excision
Soyer et al^37^	2011	12	Yes	Mass excision
Yoshimura et al^38^	1990	41	Yes	Mass excision
Mohammed et al^39^	2012	33	Yes	Mass excision
Safak et al^40^	1992	48	Yes	Mass excision
Cowen et al^41^	1957	67	Yes	Mass excision
Sighinolfi et al^42^	2006	79	Yes	Radical orchiectomy
Liu et al^43^	2021	30	Yes	Radical orchiectomy
Mahesh et al^44^	2012	26	Yes	Mass excision and scrotectomy
Abdullah et al^45^	2020	59	Yes	Mass excision
Chandrashekar et al^46^	2015	32	Yes	Mass excision
Fernandez et al^47^	1987	58	Yes	Mass excision
Schulte et al^48^	1939	49	Yes	Radical orchiectomy
Erdemir et al^49^	2008	45	Yes	Mass excision
Razzaghi et al^50^	1994	20	Yes	Mass excision
Santwani et al^20^	2010	32	Yes	Mass excision

## References

[1] Hosseini MM Geramizadeh B Shakeri S Karimi MH . Intrascrotal solitary neurofibroma: a case report and review of the literature. Urol Ann. 2012;4(2):119 121. (10.4103/0974-7796.95569) 22629013 PMC3355698

[2] Bian X Xia M Xie H , et al. Solitary testicular neurofibromatosis with testicular abscess: a case report. Transl Androl Urol. 2020;9(3):1437 1441. (10.21037/tau.2020.03.26) 32676428 PMC7354315

[3] Kazarian AG West JM Brown JA Erickson BA Gellhaus PT . Large para-testicular intra-scrotal malignant peripheral nerve sheath tumor managed with radical penectomy: a case report. Urol Case Rep. 2021;38:101695. (10.1016/j.eucr.2021.101695) 33996500 PMC8102162

[4] Hilton DA Muller S Macpherson DS . Testicular teratoma and peripheral neurofibromatosis. Postgrad Med J. 1990;66(781):974 975. (10.1136/pgmj.66.781.974) 2125127 PMC2429758

[5] Safak M Baltaci S Yaman S Uluoğlu O Eryilmaz Y . Intrascrotal extratesticular malignant schwannoma. Eur Urol. 1992;21(4):340 342. (10.1159/000474870) 1459159

[6] Gupta S Gupta R Singh S Pant L . Solitary intrascrotal neurofibroma: a case diagnosed on aspiration cytology. Diagn Cytopathol. 2011;39(11):843 846. (10.1002/dc.21558) 21994196

[7] Page MJ Moher D Bossuyt PM , et al. PRISMA 2020 explanation and elaboration: updated guidance and exemplars for reporting systematic reviews. BMJ. 2021;372:n160. (10.1136/bmj.n160) 33781993 PMC8005925

[8] Palleschi G Carbone A Cacciotti J , et al. Scrotal extratesticular schwannoma: a case report and review of the literature. BMC Urol. 2014;14:32. (10.1186/1471-2490-14-32) 24776090 PMC4030735

[9] Belakhoua SM Rodriguez FJ . Diagnostic pathology of tumors of peripheral nerve. Neurosurgery. 2021;88(3):443 456. (10.1093/neuros/nyab021) 33588442 PMC7884141

[10] Aslan S Eryuruk U Ogreden E Tasdemir MN Cınar I Bekci T . Intrascrotal extratesticular schwannoma: a rare cause of scrotal Mass. Curr Med Imaging. 2023;19(10):1210 1213. (10.2174/1573405618666220930151519) 36200252

[11] Philips S Nagar A Dighe M Vikram R Sunnapwar A Prasad S . Benign non-cystic scrotal tumors and pseudotumors. Acta Radiol. 2012;53(1):102 111. (10.1258/ar.2011.110185) 22025740

[12] Henley JD Ferry J Ulbright TM . Miscellaneous rare paratesticular tumors. Semin Diagn Pathol. 2000;17(4):319 339. 11202548

[13] Chan PT Tripathi S Low SE Robinson LQ . Case report--ancient schwannoma of the scrotum. BMC Urol. 2007;7:1. (10.1186/1471-2490-7-1) 17244372 PMC1783662

[14] Kim YJ Kim SD Huh JS . Intrascrotal and extratesticular multiple schwannoma. World J Mens Health. 2013;31(2):179 181. (10.5534/wjmh.2013.31.2.179) 24044115 PMC3770855

[15] Alsunbul A Alenezi M Alsuhaibani S AlAli H Al-Zaid T Alhathal N . Intra-scrotal extra-testicular schwannoma: a case report and literature review. Urol Case Rep. 2020;32:101205. (10.1016/j.eucr.2020.101205) 32373470 PMC7191215

[16] Arciola AJ Golden S Zapinsky J Fracchia JA . Primary intrascrotal nontesticular schwannoma. Urology. 1985;26(3):304 306. (10.1016/0090-4295(85)90135-9) 4035850

[17] Bergeron M Bolduc S Labonté S Moore K . Intrascrotal extratesticular schwannoma: a first pediatric case. Can Urol Assoc J. 2014;8(3-4):E279 E281. (10.5489/cuaj.1823) 24839501 PMC4001662

[18] Boto J Boudabbous S Lobrinus JA Gourmaud J Terraz S . Solitary neurofibroma of the spermatic cord: a case report. J Radiol Case Rep. 2015;9(6):19 28. (10.3941/jrcr.v9i6.2206) 26622934 PMC4638375

[19] Ikari R Okamoto K Yoshida T Johnin K Okabe H Okada Y . A rare case of multiple schwannomas presenting with scrotal mass: a probable case of schwannomatosis. Int J Urol. 2010;17(8):734 736. (10.1111/j.1442-2042.2010.02581.x) 20604815

[20] Bhanvadia V Santwani P . Intrascrotal extratesticular schwannoma. J Cytol. 2010;27(1):37 39. (10.4103/0970-9371.66696) 21042535 PMC2964850

[21] Issa MM Yagol R Tsang D . Intrascrotal neurofibromas. Urology. 1993;41(4):350 352. (10.1016/0090-4295(93)90594-z) 8470322

[22] Levant B Chetlin MA . Neurofibroma of tunica albuginea testis. J Urol. 1948;59(6):1187 1189. (10.1016/S0022-5347(17)69497-7) 18858064

[23] Milathianakis KN Karamanolakis DK Mpogdanos IM Trihia-Spyrou EI . Solitary neurofibroma of the spermatic cord. Urol Int. 2004;72(3):271 274. (10.1159/000077130) 15084777

[24] Pinilla I Reinoso J González-Peramato P Aguilera A de Agueda S Nistal M . Testicular albuginea neurofibroma: findings at ultrasonography and magnetic resonance imaging with pathological correlation. Arch Esp Urol. 2009;62(6):498 501. 19959867

[25] Pujani M Agarwal C Chauhan V Kaur M . Scrotal extratesticular schwannoma: a common tumor at an uncommon location. J Postgrad Med. 2018;64(3):192 193. (10.4103/jpgm.JPGM_430_17) 29992916 PMC6066616

[26] Shahid M Ahmad SS Vasenwala SM Mubeen A Zaheer S Siddiqui MA . Schwannoma of the scrotum: case report and review of the literature. Korean J Urol. 2014;55(3):219 221. (10.4111/kju.2014.55.3.219) 24648879 PMC3956953

[27] Türkyilmaz Z Sönmez K Karabulut R , et al. A childhood case of intrascrotal neurofibroma with a brief review of the literature. J Pediatr Surg. 2004;39(8):1261 1263. (10.1016/j.jpedsurg.2004.04.026) 15300541

[28] Livolsi VA Schiff M . Myxoid neurofibroma of the testis. J Urol. 1977;118(2):341 342. (10.1016/s0022-5347(17)58002-7) 894827

[29] Mahobia DHS Shrivastava DM Soni DA Sane DN Sinha DS . Perineo-scrotal schwannoma: a rare case report. Int J Surg Sci. 2018;2(4):23 25. (10.33545/surgery.2018.v2.i4a.42)

[30] Gkikas C Ram M Tsafrakidis P . Latent progression pediatric scrotal schwannoma. a case report. Urol Case Rep. 2016;6:21 23. (10.1016/j.eucr.2015.12.012) 27169021 PMC4855906

[31] Barde NG Sacchidanand S Madura C Chugh V . Intrascrotal non-testicular schwannoma: a rare case report. J Cutan Aesthet Surg. 2013;6(3):170 171. (10.4103/0974-2077.118434) 24163540 PMC3800298

[32] Bhide SP Joshi SR . Intrascrotal extratesticular neurofibroma. Annals Pathol Lab Med. 2019;6(2):C19 C21. (10.21276/APALM.2316)

[33] Mishra VC Kumar R Cooksey G . Intrascrotal neurofibroma. Scand J Urol Nephrol. 2002;36(5):385 386. (10.1080/003655902320783926) 12487746

[34] Shamsa A Omidi AA . Huge primary intrascrotal schwannoma: a case report and review of the literature. Med J Islam Repub Iran. 2004;18(1):85 86.

[35] Shukla DA Pukar DM . Rare case of scrotal schwannoma. Int J Adv Res Dev. 2017;2(12):23 26.

[36] Singal S Sekhon A Singal R Singal R . Neurofibroma of the scrotum: an unbelievable experience. Ann Trop Med Public Health. 2012;5(4):370 370. (10.4103/1755-6783.102063)

[37] Soyer T Vargel I Ayva S Cavuşoğlu T Cesur O Bülbül S . Intrascrotal extratesticular neurofibroma as a possible cause of failed descent in ipsilateral testis. Indian J Pediatr. 2012;79(1):117 119. 21617903 10.1007/s12098-011-0473-2

[38] Yoshimura K Maeda O Saiki S Kuroda M Miki T Usami M . Solitary Neurofibroma of Scrotum. J Urol. 1990;143(4):823. 2313818 10.1016/s0022-5347(17)40109-1

[39] Mohammed RH Hussein YR . Intrascrotal multiple neurofibromas unassociated with neurofibromatosis: a case report. Int J Surg Pathol. 2013;21(6):632 634. (10.1177/1066896912467372) 23204034

[40] Safak M Baltaci S Ozer G Türkölmez K Uluoğlu O . Long-term outcome of a patient with intrascrotal extratesticular malignant schwannoma. Urol Int. 1998;60(3):202 204. (10.1159/000030254) 9644798

[41] Cowen R . Tumor of the tunica vaginalis testis: case report of neurilemmoma. J Urol. 1957;77(1):59 61. (10.1016/S0022-5347(17)66522-4) 13399091

[42] Sighinolfi MC Mofferdin A De Stefani SS , et al. Benign intratesticular schwannoma: a rare finding. Asian J Androl. 2006;8(1):101 103. 16372126 10.1111/j.1745-7262.2006.00067.x

[43] Liu D Mu Y Chen P , et al. Rare primary malignant peripheral nerve sheath tumor of the left testis: a case report. Mol Clin Oncol. 2021;15(1):144. 34094542 10.3892/mco.2021.2306 PMC8165690

[44] Mahesh UK Yelikar BR Pande P Patil M . A rare case of ancient schwannoma of scrotum. Int J Biomed Adv Res;3(8). (10.7439/ijbar.v3i8.649)

[45] Abdullah Xing JP . A case report of solitary neurofibroma of the vas deferens. Urol case rep. 2020;28:101057. 10.1016/j.eucr.2019.101057 PMC6861589 31763167

[46] Chandrashekar T Kothandaraman U Lokanadham S . Rare site and size of a neurofibroma: a case report. Int Surg J. 2015;2(1):88 90. (10.5455/2349-2902.isj20150218)

[47] Fernandez MJ Martino A Khan H Considine TJ Burden J . Giant neurilemoma: unusual scrotal mass. Urology. 1987;30(1):74 76. (10.1016/0090-4295(87)90579-6) 3603916

[48] Schulte TL McDonald JR Priestley JT . Tumors of the spermatic cord: report of a case of neurofibroma. J Am Med Assoc. 1939;112(23):2405 2406. (10.1001/jama.1939.02800230029010)

[49] Erdemir F Parlaktas BS Uluocak N Filiz NO Acu B . Intrascrotal extratesticular neurofibroma: a case report. Marmara Med J. 2008;21(1):64 67.

[50] Razzaghi MR Honarmand AR Rafii MR . Neurofibromatosis presenting as scrotal elephantiasis. Med J Islam Repub Iran. 1994;8(2):137 139.

